# Lifetime Exposure to Suicide and Current Use of Suicide Prevention Competencies Among Catholic Clergy in Nigeria: A Multidimensional Structural Analysis

**DOI:** 10.1007/s10943-026-02754-8

**Published:** 2026-08-20

**Authors:** Oluchi Miracle Osondu, Lawrence Ejike Ugwu, Chijioke Kenneth Ayogu, Nwando Maryann Morah, Doris Chinelo Eyisi, Obinna Osita Ike, Jennifer E. Johnson, JohnBosco Chika Chukwuorji

**Affiliations:** 1 Department of Psychology, University of Nigeria, Nsukka, Enugu State Nigeria; 2 School of Education, Loyola Marymount University, Los Angeles California, USA; 3 Social Science Department, Faculty of Humanities, North-West University Mafikeng, Mafikeng, South Africa; 4 Charles Stewart Mott Department of Public Health, College of Human Medicine, Michigan State University, Flint, MI USA; 5 Department of Psychology, University of Pretoria, Hatfield, 0028 South Africa; 6 Promotion of Health and Innovation for Well-Being (PHI-WELL), Department of Psychology, University of Stavanger, Stavanger, Norway

**Keywords:** Community mental health, Gatekeeper role, Interprofessional collaboration, Pastoral care, Suicide prevention training

## Abstract

Despite the evidence of clergy encounters with both those at risk and survivors of suicide, research examining suicide exposure and the clergy’s use of suicide prevention competencies remains limited. We examined the associations between lifetime exposure to suicide (exposure to suicidal communication, direct exposure to suicide attempts/deaths, and indirect exposure to suicide attempts/deaths) and current use of suicide prevention competencies (ministerial approaches to postvention, nonjudgmental attitude toward people affected by suicide, confidence and knowledge in talking with a suicidal person, and pastoral care with a suicidal person). We also investigated whether suicide prevention training moderates these relationships. Participants were 336 Roman Catholic priests in Nigeria who completed the Suicidal Behaviour Exposure Scale and the Measure of Suicide Prevention Clergy Competencies, including an indicator assessing whether participants had received any formal training. Results showed that lifetime exposure to suicide was associated with the use of suicide prevention competencies among priests without formal suicide prevention training, but not among those with training. The observed moderation effect was more nuanced when we examined the dimensions of exposure in relation to the domains of competency use. Network analyses showed that, among the three exposure domains, exposure to suicidal communication was most prominent. On the clergy competency use side, pastoral care for a suicidal person demonstrated the strongest centrality. Integrating structured suicide prevention training with pastoral experience is therefore important for ensuring consistent and effective responses to individuals at risk.

**Supplementary Information:**

The online version contains supplementary material available at https://doi.org/10.1007/s10943-026-02754-8.

## Introduction

Suicide is a major public health concern, with more than 720,000 deaths occurring worldwide annually (WHO, 2025). Three out of every four deaths by suicide occur in the low- and middle-income countries, ranking as a top cause of death, particularly among youth (15–29 years old), where it is the third leading mortality cause (WHO, 2025). With about 11.2 deaths per 100,000 people per year, Africa has the highest regional suicide rate globally (WHO, 2022; 2023). The urgency of addressing suicide prevention in Africa is paramount due to the prevailing socioeconomic problems facing many African countries. The suicide crisis also extends far beyond individual deaths, as each loss creates a wide circle of exposure to suicide. Exposure refers to being aware of or affected by the suicidal thoughts and behaviors (STBs) of others within one’s social network (Mitchell et al., 2025). Suicide exposure is highly prevalent, with up to 135 people potentially affected by each death (Maple et al., 2022). A meta-analysis showed that about one in five people in the population has been exposed to the suicide of someone they know at some point in their lives (Andriessen et al., 2017), demonstrating the extensive social and psychological reach of suicide within communities.

Faith leaders are a unique group frequently exposed to suicide because they act as “key gatekeepers” in suicide prevention (Suicide Prevention Resource Center, 2005; U.S. Department of Health & Human Services, 2024) and are often the “first responders” to suicide emergencies within their communities (Rudolph, 2021). This exposure happens across several domains of ministry, from direct intervention with people in crisis to providing long-term support for survivors. Individuals in distress often turn to the clergy for help before seeking mental health professionals (Santiago et al., 2023; Yoo et al., 2026), and more than twice as many people with diagnosable mental health problems will see a cleric rather than a psychotherapist (Suicide Prevention Resource Center, 2005). Clergy members receive a yearly average of 2.02 requests for help from those who are either thinking about suicide; Catholic priests report significantly higher average annual contacts (3.49) than their Protestant (1.76) or Jewish (1.36) counterparts (Mason et al., 2020; Rudolph, 2021).

The higher frequency of reported contacts with those at risk of suicide among Catholic priests may indicate differences in pastoral engagement or help-seeking norms across religious traditions. While increased opportunities for disclosure (e.g., confession and regular pastoral contact at daily masses and office hours) may partly explain the disparity in contacts, they may also reflect a broader relational dynamic embedded within Catholic institutional practices and pastoral structures. Specifically, the sustained, person-centered accompaniment inherent in priest–parishioner relationships may function as an informal but structured mode of psychosocial support (Nwafor & Vandenhoeck, 2024; Sasmito & Lopez, 2020). Emerging work in pastoral and empirical theology conceptualizes such potentially therapeutic engagements as a form of relational care that integrates empathic presence, meaning-making, and spiritual guidance, sometimes described as “Pastrotherapy”—a participant-accompanied pastoral care model (Nwafor & Vandenhoeck, 2024). Pastrotherapy provides a useful pastoral care framework for interpreting the observed disparities and highlights the potential of clergy roles as sites of structured psychosocial intervention.

As frequent first responders to suicide emergencies, clergy are often the first called to support a family after a suicide loss occurs and are tasked with providing ongoing care to the secondary victims—the bereaved family and friends (Rudolph, 2021). Over the course of their careers, clergy are involved with conducting funeral service on an average of 2.26 suicide deaths (Mason et al., 2020). Catholic clergy report a higher involvement in suicide-related funerals, averaging 4.02 deaths compared to 1.86 for Protestant clergy (Mason et al., 2020; Rudolph, 2021), suggesting increased exposure that may reflect more frequent pastoral contact and structured relational practices positioning them as key points of engagement for individuals and families affected by suicide.

Exposure to suicide is a complex experience that can be categorized into three distinct facets based on how an individual encounters the suicidal behaviors of others: exposure to suicidal communication, direct exposure to suicide attempt/death, and indirect exposure to suicide attempt/death (Wolford-Clevenger et al., 2020). Exposure to suicidal communication entails gaining knowledge of another individual’s suicidal ideation, plans, or intent through their verbal or written expressions (Silverman et al., 2007a, 2007b; Wolford-Clevenger et al., 2020). This most often manifests when a person directly tells someone about their struggles or thoughts of ending their life (Mitchell et al., 2025). It also includes overhearing conversations or finding suicide-related communications like notes, emails, or voice messages. In modern contexts, this facet frequently involves seeing distressing comments or posts on social media.

Direct exposure to suicide attempt or death involves being a first-hand witness to a suicide attempt or the immediate physical aftermath of a death (Wolford-Clevenger et al., 2020). This can include seeing an attempt happen or discovering the event as it is starting or while it is still occurring (Silverman et al., 2007a, 2007b). For specific groups like clergy and first responders, direct exposure may involve being at the scene of a recent suicide, finding the body, or identifying the deceased. It also extends to seeing physical marks, such as cuts or scars, on a person who has self-harmed (Survivors of Suicide & Inc., 2006).

In contrast, indirect exposure to suicide attempt or death refers to learning about a suicide attempt or death through secondary channels rather than first-hand experience (Wolford-Clevenger et al., 2020). Individuals may find out about the event because someone else told them, or they may receive official notification after the fact (Defense Suicide Prevention Office, 2016; Silverman et al., 2007a, 2007b). This facet also includes exposure through media and news coverage, which can sometimes lead to suicide contagion if reporting guidelines are not followed (American Public Health Association, 2021; Substance Abuse & Mental Health Services Administration, 2016). Furthermore, indirect exposure can occur through fictional media, such as television, movies, and music, or by learning of a death within one’s broader community, such as a workplace, school, or house of worship (National Action Alliance for Suicide Prevention, 2016; National Collaborating Centre for Mental Health, 2018). Conceptualizing suicide exposure as comprising these three distinct facets recognizes that individuals encounter suicidality through qualitatively different experiences that may have distinct implications for attitudes, psychological responses, and prevention behaviors. One of such responses or behaviors may be the use of suicide prevention competencies.

Mason and colleagues (2020) have identified multiple core suicide prevention competencies specifically for clergy that support the clergy in fulfilling the gatekeeper and first responder roles. These competencies are grouped into four main domains: namely, nonjudgmental attitude toward people affected by suicide, pastoral care with a suicidal person, confidence and knowledge in talking with a suicidal person, and ministerial approaches to postvention. *Nonjudgmental attitude toward people affected by suicide* reflects an internal and communal readiness through grounded theological reflection on suicide, self-care for the leader, and building a community where congregants watch over one another (Mason et al., 2020). *Pastoral care with a suicidal person* is the skill of listening humbly and maintaining a clear identity as a spiritual leader rather than a mental health professional (Mason et al., 2020).

Intervention skills of conducting risk assessments, knowing the right questions to ask, and understanding where to refer individuals for professional help are represented as *confidence and knowledge in talking with a suicidal person* (Mason et al., 2020). Mason et al. (2020) believe that *ministerial approaches to postvention* enable clergy to appropriately minister to survivors, conduct helpful memorial services, and manage the risk of suicide contagion or clusters within the community. The multidimensional competencies imply that the application of suicide prevention competencies consists of distinct but related behavioral domains, each representing a different type of preventive action. Each of these skills may be used by the clergy.

## Suicide Exposure and Use of Suicide Prevention Competencies

In broader healthcare and first responder settings, exposure is a common reality that shapes professional readiness. Behavioral health professionals who had experienced a patient’s death by suicide showed a positive relationship with both suicide knowledge and confidence in future interventions (Wakai et al., 2020). While exposure provides the opportunity to practice skills, it does not always equate to high confidence. In a study, for instance, over half of surveyed practitioners felt “well prepared,” but a much smaller percentage (12–15%) reported feeling truly confident in their skill preparation to assess suicide risk (Schmidt, 2016). Studies of first responders highlight that frequent exposure to the suicidal behavior of others puts them at a higher risk for secondary trauma, which can complicate their ability to use competencies effectively without proper support systems (Maple et al., 2022; Wolford-Clevenger et al., 2020).

Research focusing on clergy demonstrates a significant positive association between exposure to suicide and the utilization of suicide prevention competencies. A study of 498 US clergy found that the annual number of contacts from suicidal people significantly predicted the use of a wide range of suicide prevention competencies (Mason et al., 2020). Mason et al. found that clergy reported utilizing more competencies when they were presented with increased opportunities to care for individuals in crisis, and clergy who reported higher numbers of lifetime involvements with suicide deaths also demonstrated higher levels of competence in specific domains, such as being nonjudgmental, talking about suicide in a balanced way, maintaining a clear pastoral role, and conducting helpful suicide funerals. These are often skills used across a range of care situations and are easily applied during suicide interventions.

### Theoretical Bases

The developmental model of clergy engagement with suicide (Mason et al., 2019) proposes that clergy involvement in suicide prevention originates from their roles as gatekeepers responsible for upholding the sanctity of human life and is subsequently shaped by exposure to suicide-related experiences and the learning derived from them. In other words, engagement in suicide prevention is contingent upon both role conceptualization and exposure-driven learning. While clergy recognize their moral duty to help save lives, encounters with suicidal individuals, suicide attempts, or suicide deaths within congregations may heighten clergy awareness of suicide as a pastoral concern and prompt reflection on their preparedness to respond. Consequently, suicide exposure may be associated with both the development and the use of suicide prevention competencies in pastoral practice.

According to the contact hypothesis (McKeown & Dixon, 2017) meaningful personal contact with stigmatized experiences can reduce stigma and increase empathy. In the context of suicide, contact may lead to compassion activation, whereby exposure reduces negative stereotypes about discussing suicide and may be associated with increased openness to suicide prevention training and use of supportive behaviors. However, there is also desensitization risk—some individuals may become emotionally numb or normalize suicidal behavior, reducing engagement in preventative actions (Cantor, 2003).

Based on Theory of Planned Behavior (Ajzen, 1991), exposure may shift attitudes (seeing prevention as more important), heighten normative support (others expect you to act), and increase perceived control (confidence gained from experience). The theory postulates that the frequency of suicide exposure significantly impacts the use of prevention competencies, primarily through a process of experiential or “on-the-job” learning. A study by Mason and colleagues (2021) suggests that contact with individuals experiencing suicidal thoughts may increase clergy interest in and receptivity to suicide prevention training. In a large sample of US clergy, majority reported annual contact with individuals experiencing suicidal ideation and also expressed a need for more training, indicating a link between exposure and perceived training needs (Mason et al., 2021). Still, exposure does not guarantee positive outcomes—it can also lead to distress, avoidance, or stagnation. In environments where suicide is highly stigmatized or predominantly construed through moral or theological frameworks, disclosure to clergy may be limited, thereby constraining opportunities for experiential learning. This suggests that exposure may be systematically attenuated even under conditions of high population-level risk. Moreover, competing role expectations, institutional priorities, and structurally limited access to training may further inhibit the translation of epidemiological need into professional motivation. In other words, due to a number of moderating factors, exposure to suicide may have divergent effects, simultaneously fostering compassion and risking desensitization. Hence, the contributions of exposure to competency use may be conditional to other factors.

### Training as a Moderator Variable

Moderators that may either strengthen or weaken the association between exposure and use of suicide prevention competencies include personal processing of the experience, support systems available, and training and reflective opportunities before or after exposure. Mason et al. (2020) reported that while contact with suicidal individuals predicts competency use, suicide-specific training was the other critical, independent predictor of a professional’s ability to intervene accurately. Most clergy develop suicide prevention competency skills “on the job” rather than through formal seminary training (Mason et al., 2020), highlighting the importance of formal suicide prevention training and experience. While mental health training programs for professionals insufficiently address suicide-related training (Mitchell et al., 2020; Schmitz et al., 2012), we observed that such formal suicide prevention training is currently lacking in seminary education and pastoral formation in Nigeria. Despite the training gaps, clergy continue to encounter individuals with suicide risk or attempt survivors, engage survivors of suicide loss, and conduct funerals following suicide-related death. The significant gaps in clergy preparation may influence the link between suicide exposure and current use of suicide prevention competencies.

### The Present Study

Research examining how suicide exposure influences clergy’s use of suicide prevention competencies remains limited. Except for Mason et al. (2020), existing studies have given limited attention to the multifaceted behavioral application of suicide prevention competencies. Yet, Mason et al.’s study did not consider the multifaceted nature of suicide exposure in relation to use of suicide prevention competencies. Mason et al. suggested that high-frequency contact and formal training together predict a higher utilization of the full range of prevention skills, but researchers have not tested the postulation that receiving formal suicide prevention training moderates the association between suicide exposure and competencies use. Furthermore, little research has explored exposure to suicide and clergy use of suicide prevention competencies in non-Western contexts, including African faith communities. Many Africans express religious sentiments on suicide (Nwafor, 2025a; Ogbolu et al., 2020; Osafo et al., 2013, 2019) and faith leaders play important roles in responding to suicide-related crises and providing therapy for mental disorders (Iheanacho et al., 2021; James et al., 2014; Nwafor, 2024; Osafo et al., 2021), yet many clergy lack formal training in suicide prevention (Nwafor, 2024, 2025b), highlighting the need for research on clergy preparedness and competencies. Our study aims to examine the associations between lifetime exposure to suicide and the current use of suicide prevention competencies and to investigate whether suicide prevention training moderates these relationships.

### Hypotheses

We hypothesized that: (1) Lifetime exposure to suicide is positively associated with current clergy suicide prevention competencies. (2) The relationship between exposure and competency is moderated by prior suicide prevention training, such that the structural path differs between priests who indicated that they have received formal suicide prevention training and those who have not received such training. (3) The exposure–competency association is concentrated in specific domain-level linkages, identifiable in a regularized partial correlation network (GGM), rather than being diffuse across all domains.

## Method

### Participants and Procedure

Participants in this study were 336 **Roman** Catholic priests (mean age = 45.17, SD = 10.59, age range 27–78 years) purposively drawn from seven Catholic dioceses in Nigeria. With over 30 million adherents, the Roman Catholic Church is the largest Christian denomination in the country and also represents the 12th largest Catholic population worldwide and the second largest in Africa (Catholics & Cultures, n.d.; Centre for Applied Research in the Apostolate, 2023). Within this context, Catholic priests occupy influential pastoral roles and often serve as trusted sources of guidance for parishioners. All priests who participated in this study had at least two academic degrees because Catholic priests in Nigeria are required to complete their Philosophy and Theology studies prior to ordination. Their current primary roles were: pastors (or parish priests, 65.8%), pastoral vicars (17.9%), educators (5.7%), seminary formators (7.4%), and others (3.2%). Ethical approval for the study was granted by the Psychology Research Ethics Committee of the University of Nigeria, Nsukka.

Authorization to conduct the study was obtained from diocesan authorities, including Vicars for priests, diocesan secretaries or chancellors, or both. Data collection took place between June and October 2024. The first author personally administered the questionnaires to Catholic priests across several dioceses, mainly during monthly recollections—a half-day period devoted to prayer and reflection—or during annual retreats, which typically involve a five-day withdrawal from regular activities for deeper spiritual renewal, discernment, and reflection on one’s life and ministry. At these gatherings, the first author was formally introduced, after which the purpose of the study was explained, and priests were invited to complete the paper-and-pencil questionnaire. Participants were assured that their responses would remain anonymous and confidential, that participation was voluntary, and that they could withdraw from the study at any time without any consequences. No financial incentives were offered. The priests were encouraged to respond honestly to the questionnaire items, which included clear instructions for completion. Completed questionnaires were collected immediately after completion. The study achieved an overall response rate of 86%, indicating a high level of engagement among priests across the participating dioceses.

### Instruments

Two instruments were used in this study: The Suicidal Behaviour Exposure Scale (SBX scale, Wolford-Clevenger et al., 2019, 2020) and the Measure of Suicide Prevention Clergy Competencies (MSPCC, Mason et al., 2020). The questionnaire also included a section for relevant demographic information and some other questions with either dichotomous response or open-ended response options. To measure the formal suicide prevention training received, we asked “*Have you had any training on the topic of suicide prevention?*” [Yes/No]. We included three additional items to measure clergy experiences with suicidal individuals and their unique pastoral roles. The priests were asked: “*How many persons thinking about suicide request your help annually?*” and “*How many persons who have attempted suicide request your help annually?*” To assess pastoral involvement in suicide prevention-related deaths, they were asked: “*Annually, how many suicide-related funerals do you conduct?*”.

#### Suicidal Behavior Exposure Scale (SBX Scale, Wolford-Clevenger et al., 2019, 2020)

The SBX scale (Wolford-Clevenger et al., 2019, 2020) evaluates different types of exposure to suicide-related thoughts and behaviors. In the development process, Wolford-Clevenger and colleagues adopted definitions of exposure by the Suicide Nomenclature Work Group (Silverman et al., 2007a, 2007b) encompassing exposure to suicidal ideation, threats, attempts, and fatalities and analyzed the Modified Scale for Suicidal Ideation (Miller et al., 1986) to discern numerous events associated with suicidal thoughts that individuals may encounter. Subsequent items were developed based on these occurrences, integrating both indirect (e.g., hearsay) and direct (e.g., observation) exposure to suicide threats, attempts, and fatalities. The scale was evaluated by the developers for content validity, culminating in a 16-item instrument with three dimensions exposure to suicidal communication (e.g., heard/seen someone wish for death/suicide), direct exposure to suicide attempts/deaths (e.g., witnessed someone’s death by suicide), and indirect exposure to suicide attempts/deaths (e.g., been told specific details about someone’s dying by suicide). The scale evaluates exposure frequency using a 5-point Likert scale (0 = never to 4 = very often). Higher scores on each of the dimensions also reflect higher exposure to suicide on the specific aspect. The internal consistencies (Cronbach’s *α*) were as follows: 0.79 (exposure to suicidal communication), 0.89 (direct exposure to suicide attempts/deaths), and 0.64 (indirect exposure to suicide attempts/deaths) (Wolford-Clevenger et al., 2019). For the current sample, Cronbach’s *α* were 0.78, 0.71, and 0.69, for exposure to suicidal communication, indirect exposure to suicide attempt/death, and direct exposure to suicide attempt/death, respectively.

#### Measure of Suicide Prevention Clergy Competencies (Mason et al., 2020)

The Measure of Suicide Prevention Clergy Competencies (MSPCC) (Mason et al., 2020) comprises 17 competencies, each transforming into an action statement reflecting a practical stage of clergy competence in suicide prevention. For example, the competency “Knowing role as clergy” at the practical stage is represented as “Keeps identity pastoral,” which is converted to the item: “When a suicidal person talks to me, I am clear about my role as a spiritual leader, not a mental health professional.” Mason et al. (2020) reported a four-factor structure of the MSPCC as follows: ministerial approaches to postvention [e.g., *When a suicide death happens, I know how to conduct a funeral that is helpful to survivor(s) and congregant(s)*], **nonjudgmental** attitude toward people affected by suicide [e.g., *When a person who has attempted suicide talks to me, I am nonjudgmental*], Confidence and knowledge in talking with a suicidal person [e.g., *When a suicidal person talks to me, I know the right questions to ask*], and pastoral care with a suicidal person [e.g., *When a suicidal person talks to me, I am clear about my role as a spiritual leader not a mental health professional*]. Respondents use a 5-point scale ranging from “never/not like me” (0) to “always/like me” (4). Internal consistency reliability (Cronbach’s *α*) for the overall measure is 0.85. The dimensions have αs of 0.82 (ministerial approaches to postvention), 0.96 (nonjudgmental attitude toward people affected by suicide), 0.81 (confidence and knowledge in talking with a suicidal person), and 0.46 (pastoral care with a suicidal person). For the current sample, αs were 0.83, 0.83, 0.88, and 0.87, for ministerial approaches to postvention, nonjudgmental attitude toward people affected by suicide, confidence and knowledge in talking with a suicidal person, and pastoral care with a suicidal person, respectively.

### Statistical Analyses

Responses on the SBX scale and the MSPCC were checked for out-of-range values; erroneous entries were recoded as missing (final analytic *N* = 336). Dimension scores were computed as item means and rescaled to the original metric to minimize loss of information due to occasional item nonresponse. Descriptive statistics and Pearson correlations were computed for all continuous variables. Primary hypotheses were tested using structural equation modeling (SEM) in *R*. SEM and multigroup SEM models were estimated with the lavaan package using maximum likelihood estimation and full-information maximum likelihood (FIML) for missing data. Exposure to suicide was modeled as a latent construct indicated by exposure to suicidal communication, direct exposure, and indirect exposure, while suicide prevention competencies were modeled as a latent construct indicated by **nonjudgmental** attitude toward people affected by suicide, pastoral care with a suicidal person, confidence and knowledge in talking with a suicidal person, and ministerial approaches to postvention. Model fit was evaluated using χ^2^, CFI, TLI, RMSEA (90% CI), and SRMR.

Moderation of the association between lifetime suicide exposure and current use of suicide prevention competencies by prior suicide prevention training was examined via multigroup SEM (MG-SEM) (clergy who endorsed that they have received prior suicide prevention training vs those without such training) by comparing a freely estimated model with a model constraining structural paths to equality across groups using a likelihood ratio test (Δχ^2^). Age, years since ordination, annual encounters with suicidal ideation, annual encounters with suicide attempts, and suicide prevention-related funerals were included as covariates predicting competencies within each group. To address domain-specific moderation, follow-up multigroup path models were estimated in lavaan, specifying simultaneous effects of the three exposure dimensions on the four competency domains (with the same covariates). Group differences in regression paths were tested using Δχ^2^, and significant effects were summarized using group-specific simple slopes. Finally, domain-level mechanisms were examined using a regularized Gaussian graphical model (GGM) estimated in *R* (via bootnet/qgraph with EBICglasso regularization). Expected influence centrality quantified node importance, and edge weights represented regularized partial correlations. Network stability and accuracy were evaluated using case-dropping and nonparametric bootstrap procedures (1000 iterations each).

## Results

As shown in Table 1, the SBX dimensions were moderately intercorrelated (*r* = 0.33–0.56), whereas the MSPCC domains were strongly intercorrelated (*r* = 0.40–0.64). Across constructs, exposure indicators showed small-to-moderate positive associations with competency domains (approximately *r* = 0.13–0.23), consistent with the expectation that greater lifetime exposure to suicide is associated with greater reported use of suicide prevention competencies.

**Table 1:** Means, standard deviations, and bivariate correlations for all continuous variables

Variable	*M*	SD	1	2	3	4	5	6	7	8	9	10	11
1. Age	45.17	10.59	–										
2. Priestly work experience (years)	13.42	10.38	0.93^**^	–									
3. Suicidal ideation encounters	21.61	3.95	0.10	0.12^*^	–								
4. Suicide attempt encounters	0.84	1.31	0.10	0.12^*^	0.72^**^	–							
5. Suicide-related funeral	0.24	0.65	0.05	0.05	0.05	0.11^*^	–						
6. Exposure to SC	9.77	2.90	− 0.08	− 0.08	0.16^**^	0.19^**^	0.12^*^	–					
7. Indirect exposure	7.50	2.64	− 0.07	− 0.04	0.09	0.09	0.13^*^	0.52^**^	–				
8. Direct exposure	8.08	2.57	− 0.04	− 0.02	0.07	0.11^*^	0.07	0.56^**^	0.33^**^	–			
9. Nonjudgmental attitude	5.82	2.84	0.05	0.07	0.05	0.13^*^	0.13^*^	0.18^**^	0.22^**^	0.06	–		
10. Pastoral care	11.30	3.60	0.12^*^	0.12^*^	0.20^**^	0.17^**^	0.04	0.19^**^	0.23^**^	0.13^*^	0.54^**^	–	
11. Confidence and knowledge	10.94	3.69	0.15^**^	0.14^*^	0.22^**^	0.14^*^	0.06	0.14^*^	0.11	0.08	0.42^**^	0.64^**^	–
12. MPA	8.91	3.45	0.12^*^	0.15^*^	0.17^**^	0.14^*^	0.07	0.13^*^	0.15^*^	0.08	0.40^**^	0.55^**^	0.54^**^

*N* = 336. Variables 6–8 are SBX dimensions. Variables 9–12 are MSPCC dimensions. ^*^p < 0.05. ^**^p < 01; Suicidal ideation encounters = Annual encounters with individuals thinking about suicide; Suicide attempt encounters = Annual encounters with individuals who attempted suicide; Suicide-related funeral = Number of suicide-related funerals conducted annually; SC, Suicidal communication; MAP, Ministerial approach to postvention

### Structural Equation Modeling

To test the primary hypothesis that lifetime exposure to suicide is associated with greater use of suicide prevention competencies among clergy, a structural equation model was estimated using maximum likelihood with full-information maximum likelihood (FIML) to account for missing data. Model fit was excellent, χ^2^(13) = 18.64, *p* = 0.135; CFI = 0.991; TLI = 0.986; RMSEA = 0.036, 90% CI [0.000, 0.070]; SRMR = 0.034.

#### Hypothesis 1:

Lifetime exposure to suicide is positively associated with current clergy suicide prevention competencies.

**Fig. 1:**
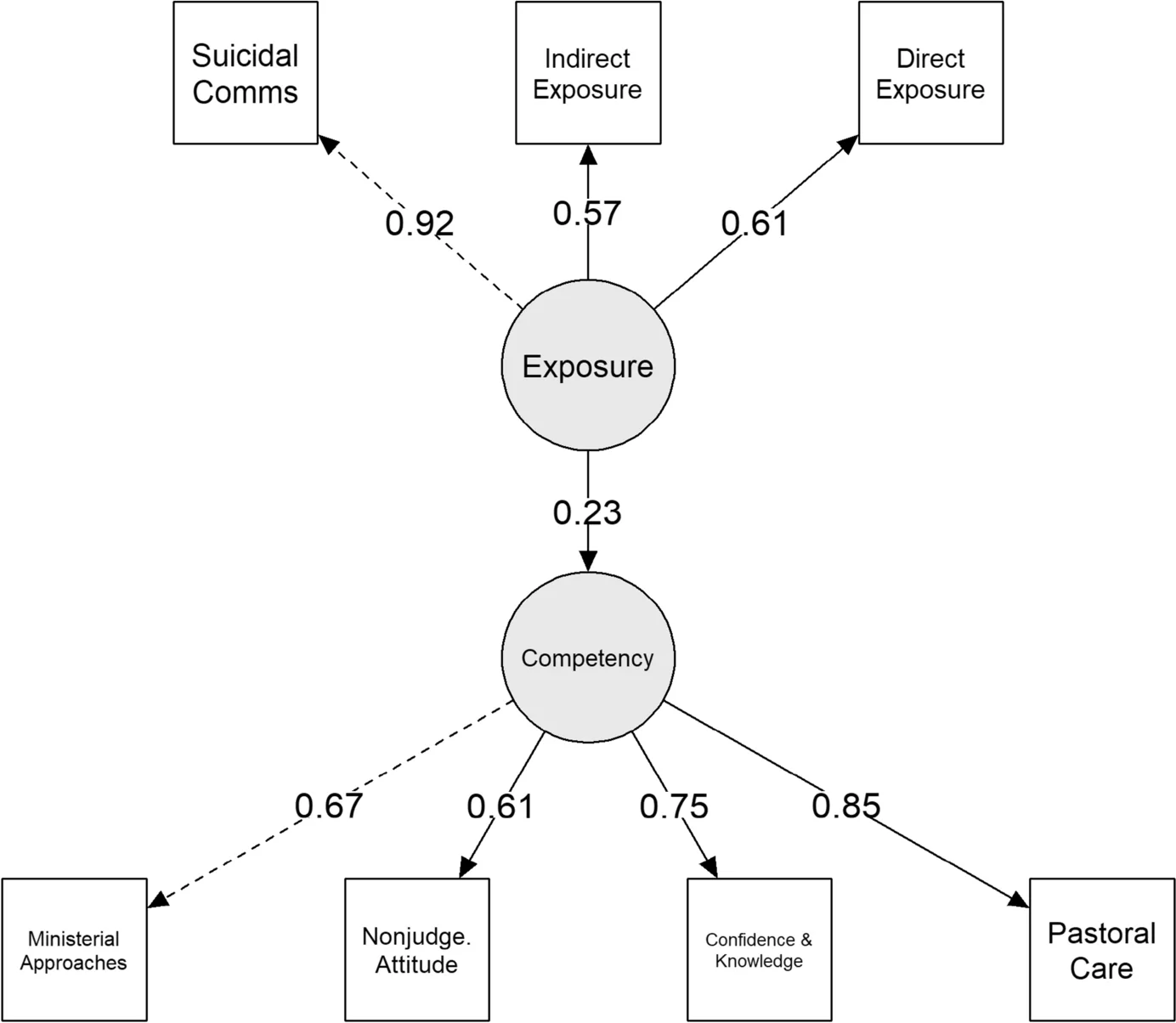
Structural equation model (SEM) path diagram

In the measurement model, indicators loaded significantly and positively onto their respective latent constructs (all *p* < 0.001; Table 2), supporting the hypothesized measurement structure for SBX (three dimensions) and MSPCC (four domains). Consistent with the primary hypothesis, lifetime exposure to suicide significantly predicted higher suicide prevention competencies (β = 0.23, *z* = 3.28, *p* = 0.001). The exposure latent factor explained a modest 5.5% of the variance in clergy competencies (*R*^2^ = 0.055), suggesting that although clergy who have more exposure to suicide cases tend to have slightly higher competencies, other factors could play substantial roles. The hypothesized latent structure and standardized pathways are depicted in Fig. 1.

**Table 2:** Standardized factor loadings for the measurement model by training group

Latent construct/indicators	Untrained group (*β*)	Trained group (*β*)	*p* value
Exposure to suicide			
Suicidal communication	0.91	0.94	< 0.001
Direct exposure	0.56	0.73	< 0.001
Indirect exposure	0.56	0.58	< 0.001
Prevention competencies			
Pastoral care	0.87	0.82	< 0.001
Confidence and knowledge	0.66	0.94	< 0.001
**Nonjudgmental** attitude	0.62	0.56	< 0.001
Ministerial approaches	0.62	0.77	< 0.001

*N* = 336 (Untrained refers to those who have never received any formal suicide prevention training, *n* = 250; Trained refers to those who endorsed receiving a formal suicide prevention training, *n* = 86). All factor loadings are standardized and significant at *p* < 0.001

#### Hypothesis 2:

The relationship between exposure and competency is moderated by prior suicide prevention training, such that the structural path differs between priests who indicated that they have received formal suicide prevention training and those who have not received such training.

To evaluate whether formal suicide prevention training moderated the exposure–competency association, a multigroup SEM (MG-SEM) was estimated comparing clergy with no prior training (*n* = 250) to clergy who had received training (*n* = 86). Five covariates (age, years since ordination, annual encounters with individuals thinking about suicide, annual encounters with individuals who attempted suicide, and number of suicide prevention-related funerals) were included as predictors of competencies in each group to isolate the unique contribution of lifetime exposure. A likelihood ratio test comparing a freely estimated model against a model constraining structural paths to equality across groups indicated significantly worse fit under equality constraints, Δχ^2^(6) = 13.74, *p* = 0.033, confirming training status as a moderator.

Among priests who have not received a formal suicide prevention training, lifetime exposure to suicide was a significant predictor of the use suicide prevention competencies (*β* = 0.32, *p* = 0.001), whereas none of the covariates were statistically significant predictors (Table 3). In contrast, among those who endorsed receiving formal suicide prevention training, lifetime exposure was not significantly associated with competencies (*β* = 0.08, *p* = 0.488). Instead, annual encounters with individuals thinking about suicide emerged as a significant positive predictor of competencies (*β* = 0.34, *p* = 0.036; Table 3).

**Table 3:** Standardized structural path coefficients predicting clergy use of suicide prevention competencies

Predictor variable	Untrained group (*n* = 250) *β*	*p*	Trained group (*n* = 86)*β*	*p*
*Primary predictor*				
Lifetime exposure to suicide	0.32	0.001	0.08	0.488
*Covariates*				
Age	0.15	0.444	− 0.06	0.851
Years since ordination	− 0.08	0.683	0.40	0.165
Suicide ideation encounters	0.14	0.116	0.34	0.036
Suicide attempt encounters	− 0.02	0.843	− 0.15	0.362
Suicide-related funerals	− 0.07	0.392	0.20	0.051

Untrained refers to those who have never received any formal suicide prevention training, while trained refers to those who endorsed receiving a formal suicide prevention training;* β* represents standardized path coefficients. Unconstrained MG-SEM fit: χ^2^(86) = 141.02, *p* < 0.001; CFI = 0.924; TLI = 0.901; RMSEA = 0.062; SRMR = 0.069

Because the primary hypothesis was specified at the latent construct level (overall exposure → overall competencies), moderation was first evaluated using MG-SEM to provide a parsimonious, measurement error-adjusted test while limiting parameter proliferation given the trained subgroup size. To address concerns that construct-level moderation could mask domain-specific differences, follow-up analyses tested moderation at the dimension level. We conducted multigroup path analysis, which examined pathways from the three SBX exposure dimensions to the four MSPCC competency domains while controlling for the same covariates as in the MG-SEM. A likelihood ratio test comparing a freely estimated model to a model constraining regression paths to equality across training groups confirmed that dimensional pathways differed significantly between those who have received a formal training in suicide prevention and those who reported not receiving such a training, Δχ^2^(32) = 71.52, *p* < 0.001.

**Fig. 2:**
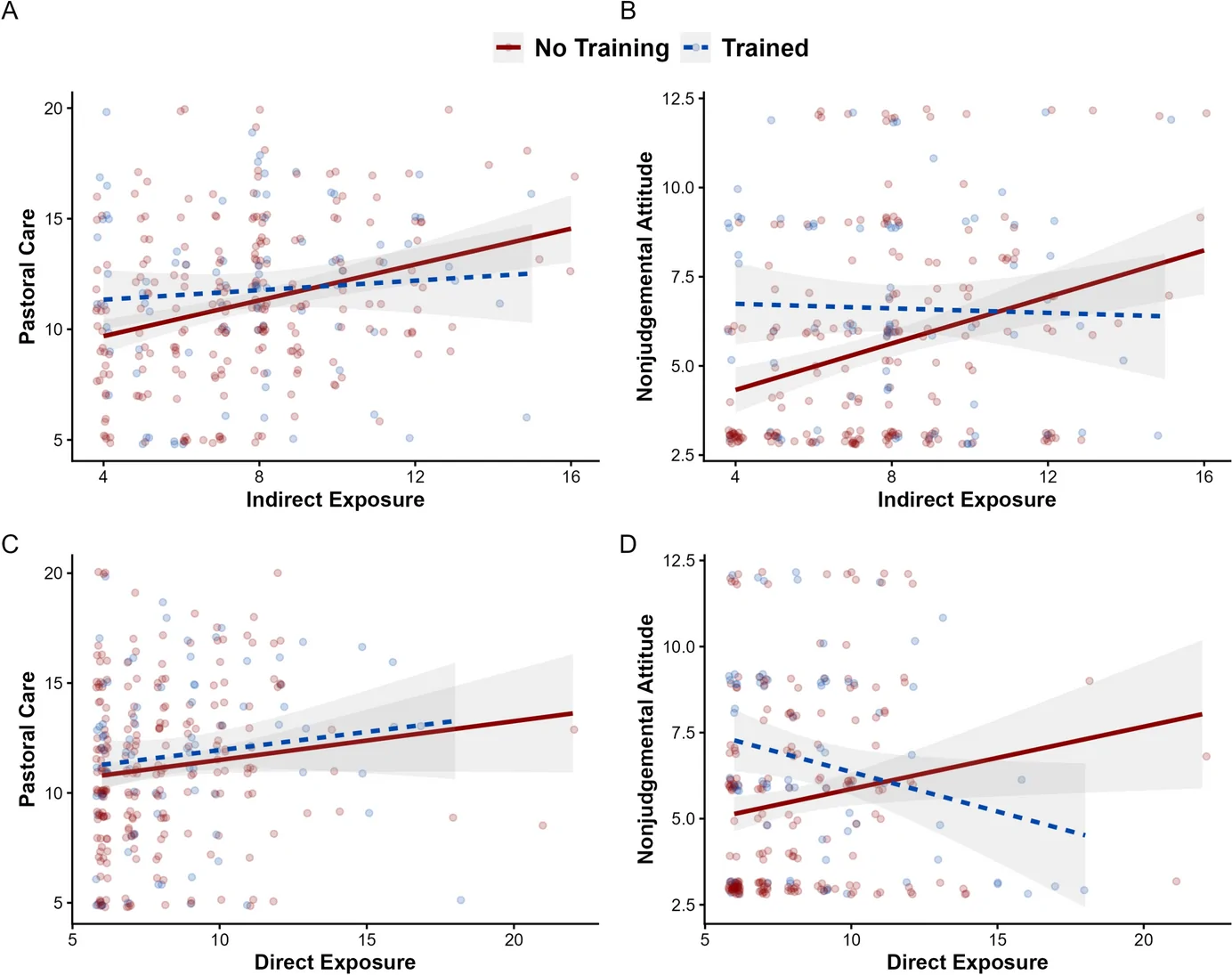
Simple slope interaction plots demonstrating the moderating effect of suicide prevention training on key exposure–competency pathways

As shown in Table 4, among those who have never received formal suicide prevention training, indirect exposure to suicide attempt/death significantly predicted pastoral care with a suicidal person (*β* = 0.22, *p* = 0.002) and nonjudgmental attitude toward people affected by suicide (*β* = 0.21, *p* = 0.005). Exposure to suicidal communication strongly predicted confidence and knowledge in talking with a suicidal person (*β* = 0.22, *p* < 0.01). Among clergy who endorsed receiving a formal suicide prevention training, indirect exposure to suicide attempt/death was not significantly associated with any competency domain (all *p* > 0.50). In the trained group, higher direct exposure to suicide attempt/death was associated with higher pastoral care with a suicidal person (*β* = 0.28, *p* = 0.039) and lower nonjudgmental attitude toward people affected by suicide (*β* =  − 0.33, *p* = 0.016). Those with higher exposure to suicidal communication reported higher pastoral care with a suicidal person (*β* = 0.35, *p* = 0.026). Higher exposure to suicidal communication was also associated with higher confidence for the trained group, but not as strongly as it was for the untrained group (Table 4). Simple-slope patterns for the moderated pathways are visualized in Fig. 2.

**Table 4:** Moderated pathways from types of suicide exposure (indirect, direct, and suicidal communication) to suicide competencies (pastoral care, nonjudgmental attitude, confidence, and ministerial approach) by training status

Predictor → Outcome	Clergy group	*B*	SE	*β*	*p* value
Indirect exposure → Pastoral care	Untrained	0.31	0.10	0.22	**0.002**
Indirect exposure → Pastoral care	Trained	0.10	0.16	0.08	0.523
Indirect exposure → Nonjudgmental attitude	Untrained	0.23	0.08	0.21	**0.005**
Indirect exposure → Nonjudgmental attitude	Trained	− 0.00	0.13	− 0.00	0.974
Direct exposure → Pastoral care	Untrained	− 0.02	0.10	− 0.02	0.823
Direct exposure → Pastoral care	Trained	0.35	0.17	0.28	**0.039**
Direct exposure → Nonjudgmental attitude	Untrained	0.04	0.08	0.03	0.669
Direct exposure → Nonjudgmental attitude	Trained	− 0.32	0.13	− 0.33	**0.016**
Exposure to suicidal communication → Confidence	Untrained	0.48	0.18	0.22	**0.008**
Exposure to suicidal communication → Confidence	Trained	0.19	0.08	0.36	**0.018**
Exposure to suicidal communication → Pastoral care	Untrained	0.16	0.17	0.07	0.345
Exposure to suicidal communication → Pastoral care	Trained	0.18	0.08	0.35	**0.026**

*N* = 336 (Untrained refers to those who have never received any formal suicide prevention training *n* = 250; Trained refers to those who endorsed receiving formal suicide prevention training, *n* = 86). *B* = unstandardized coefficient; SE, standard error;* β*, standardized coefficient. “Suicidal ideation encounters” refers to annual encounters with individuals thinking about suicide. Bold *p* values are for the significant pathways

Together, these findings indicate that training alters the domain-level pathways linking exposure and competencies: Competency use for clergy who have not received formal suicide prevention training is more strongly associated with indirect/community exposure, whereas competency use for those with formal suicide prevention training is more closely linked to exposure to suicidal communication and shows a different pattern for direct exposure across domains (Table 4; Fig. 2).

#### Hypothesis 3:

 The exposure–competency association is concentrated in specific domain-level linkages, identifiable in a regularized partial correlation network (GGM), rather than being diffuse across all domains.

**Fig. 3:**
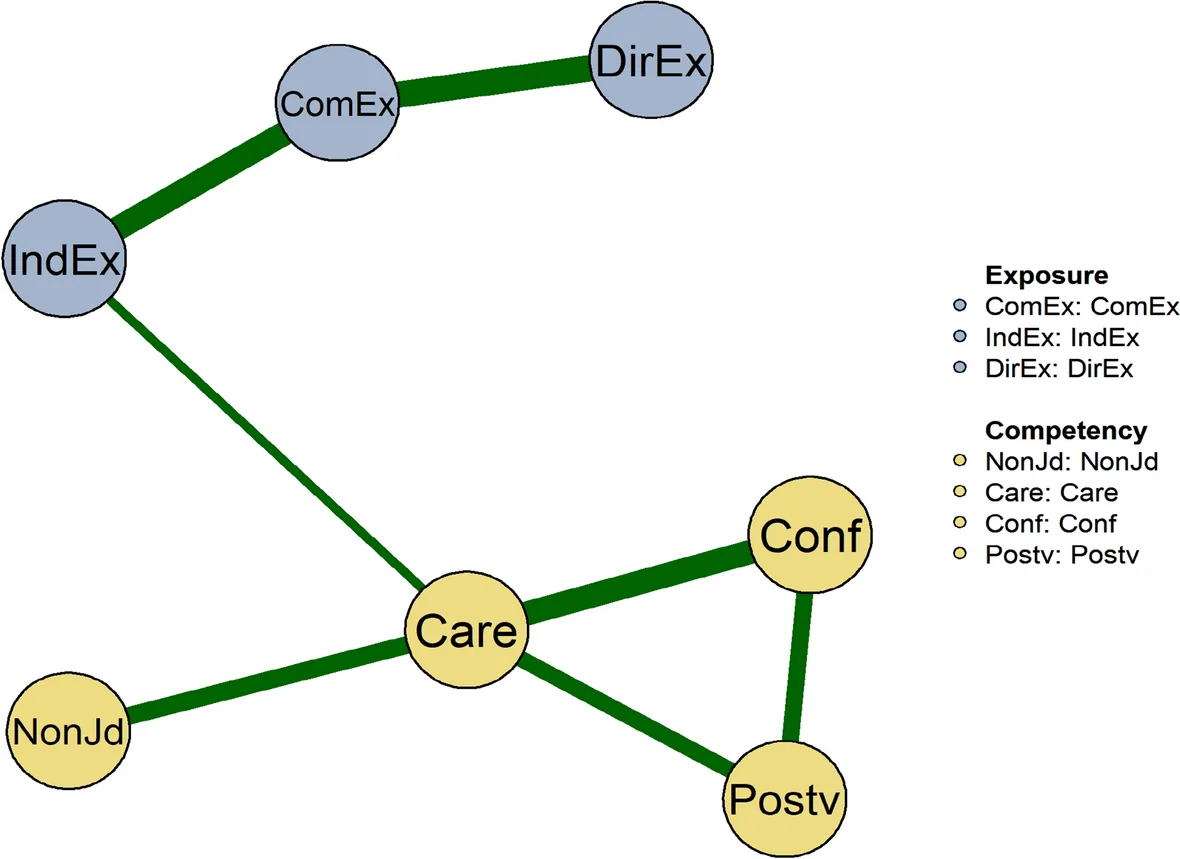
Gaussian graphical model network structure (regularized partial correlations)

**Fig. 4:**
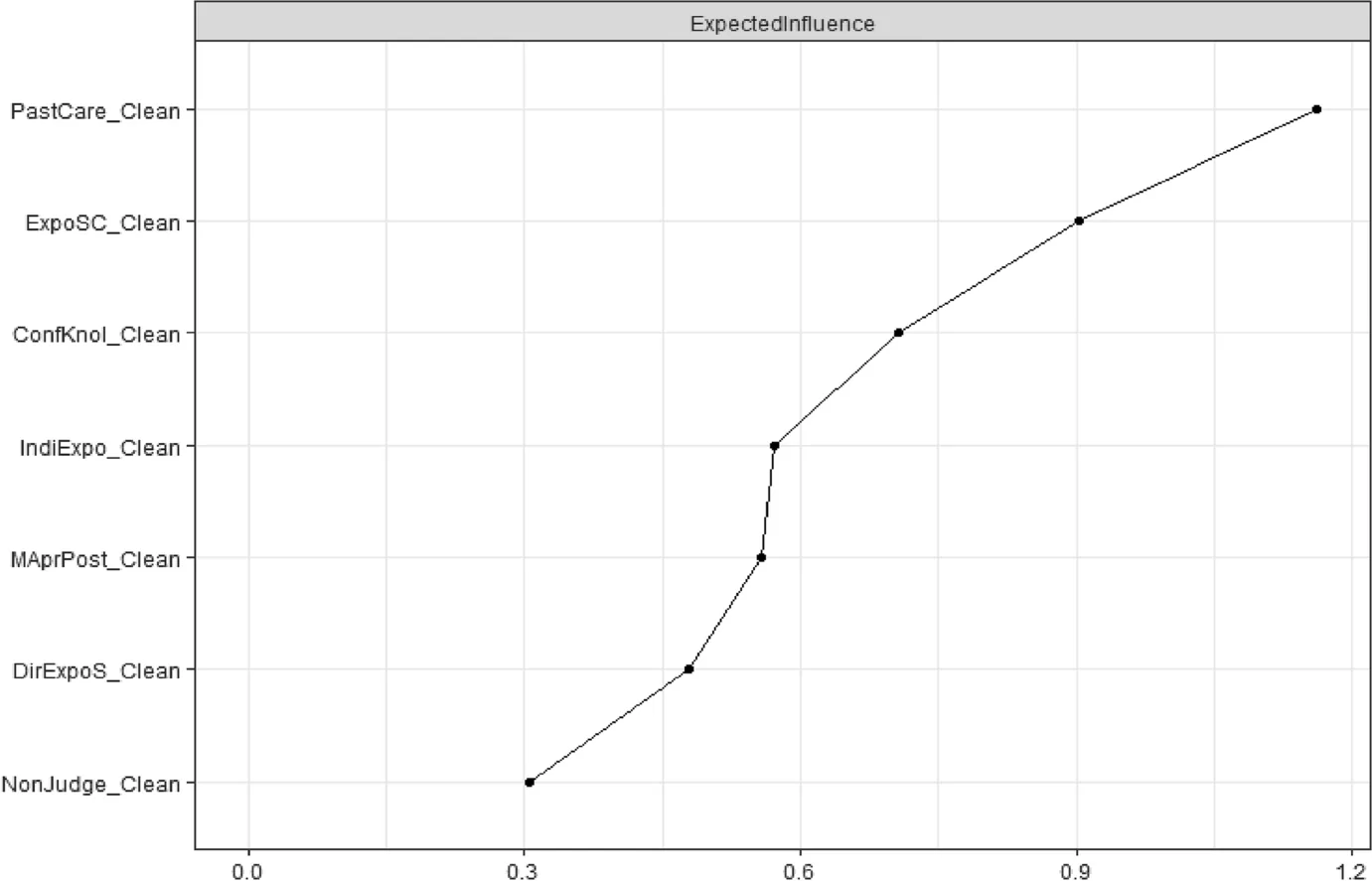
Expected influence centrality plot

To further identify which exposure and competency dimensions most strongly account for the overall exposure–competency association, SEM was complemented with a regularized GGM estimated at the domain level. The network was estimated using EBICglasso regularization to obtain a sparse partial correlation structure. Node importance was evaluated using expected influence (EI). The estimated network structure is shown in Fig. 3, and EI centrality estimates are summarized in Table 5 and Fig. 4.

**Table 5:** Standardized expected influence centrality estimates for exposure and competency dimensions

Variable category	Specific dimension	Expected influence (*z*-score)
Competency	Pastoral care	1.72
Exposure	Suicidal communication	0.82
Competency	Confidence and knowledge	0.13
Exposure	Indirect exposure	− 0.34
Competency	Ministerial approaches	− 0.39
Exposure	Direct exposure	− 0.67
Competency	**Nonjudgmental** attitude	− 1.27

Expected influence quantifies the overall strength of a node’s connections within the GGM. Higher positive values indicate stronger centrality

On the exposure side, exposure to suicidal communication exhibited the greatest centrality (standardized EI = 0.82), whereas indirect exposure to suicide attempt/death (standardized EI =  − 0.34) and direct exposure to suicide attempt/death (standardized EI =  − 0.67) were comparatively peripheral. On the competency side, pastoral care with a suicidal person emerged as the most central node in the full network (standardized EI = 1.72), followed by confidence and knowledge in talking with a suicidal person (standardized EI = 0.13), whereas nonjudgmental toward people affected by suicide was least central (standardized EI =  − 1.27).

Inspection of regularized edge weights indicated that most cross-domain partial correlations were shrunk to zero. The most prominent conditional association bridging exposure and competency domains was the indirect exposure–pastoral care edge (*r* = 0.145), which is visible in Fig. 3 and quantified in Table 6 (see Appendix in supplementary material). Notably, the bridging pattern converges with the dimensional moderation results in highlighting indirect exposure and pastoral care as a key linkage, particularly among clergy who have not received a formal suicide prevention training (see earlier results in Table 4).

#### Network Robustness and Stability

The stability of expected influence centrality was evaluated using a case-dropping bootstrap procedure (1000 iterations). Centrality estimates remained strongly correlated with the original-sample estimates across substantial proportions of removed cases, indicating good-to-excellent stability of the expected influence ordering (Fig. 5, see Appendix in supplementary material). Edge-weight accuracy was examined using a nonparametric bootstrap (1000 iterations). Confidence intervals were wider for weaker edges and narrower for stronger edges, indicating acceptable precision for the most pronounced edges, whereas very small edges showed greater uncertainty (Fig. 6, see Appendix in supplementary material). Collectively, these diagnostics support the robustness of the key centrality pattern and the strongest estimated edges.

## Discussion

Given the recognized role of clergy as key suicide prevention gatekeepers, we examined the associations between lifetime exposure to suicide and the current use of suicide prevention competencies and investigated whether suicide prevention training moderates these relationships. Overall and across all the dimensions, greater lifetime exposure to suicide was associated with higher suicide prevention competencies. This finding is consistent with our first hypothesis that lifetime exposure to suicide would be positively associated with clergy suicide prevention competencies. This new evidence also aligns with past research findings in western countries showing that cumulative encounters with suicide-related events may contribute to experiential learning in pastoral contexts (Mason et al., 2019; 2020).

Among priests who have not received formal suicide prevention training, higher lifetime exposure to suicide predicted increased use of suicide prevention competencies, whereas no significant association was observed among those with such training. This finding is consistent with our second hypothesis that the relationship between exposure and competency is moderated by prior suicide prevention training, such that the structural path differs between priests who indicated that they have received formal suicide prevention training and those who have not received such training. The exposure–competency association was most pronounced in the absence of formal suicide prevention training, corroborating the key role of training in enhancing suicide prevention competencies as highlighted previously (Mason et al., 2020).

The observed moderation effect became more nuanced when we examined the dimensions of exposure and competency use. Specifically, among priests without formal suicide prevention training, higher indirect exposure predicted greater engagement in pastoral care with a suicidal person and a more nonjudgmental attitude toward individuals affected by suicide, indicating that vicarious encounters with suicide within their social or ministerial networks may heighten awareness and foster empathic responses. None of the paths between indirect exposure to suicide attempt/death and any of the competency use domains was significant for priests who endorsed receiving a formal suicide prevention training. This finding suggests that indirect exposure to suicide attempt or death may function as an informal learning experience for priests who lack formal suicide prevention training. We believe that the absence of significant associations of indirect exposure to suicide attempt or death and competencies use among priests who had received formal suicide prevention training is because training may standardize or equalize competency levels, reducing the extent to which experiential exposure predicts variations in pastoral responses among the subgroup.

Among priests who reported receiving suicide prevention training, higher direct exposure to suicide attempt or death predicted greater engagement in pastoral care with a suicidal person. This suggests that formal suicide prevention training may enhance the practical application of experiences with suicide in pastoral care. Training may equip clergy with the skills needed to translate direct encounters with suicide-related crises into more active and supportive pastoral responses. While indirect exposure may prompt learning and compassionate engagement among priests who have not received formal suicide prevention training, reliance on such experiences alone is inconsistent and potentially insufficient for developing comprehensive suicide prevention competencies.

Generally, formal training may provide a more systematic foundation for pastoral care, ensuring that priests are equipped with consistent knowledge and skills for responding effectively to suicidal individuals and supporting those affected by suicide. The training may enable priests to more effectively utilize their real-life experiences with suicide-related events when providing care to individuals in distress. In other words, when training is present, direct exposure may reinforce rather than overwhelm clergy, allowing them to respond with greater pastoral engagement and competence. This finding supports the urgent necessity of integrating structured suicide prevention training into clergy formation and continuing pastoral education in Nigeria so that clergy can respond more effectively when confronted with suicide-related situations in their ministries.

Our third hypothesis which proposed that the association between exposure and competency would be concentrated within specific domain-level linkages rather than distributed diffusely across all domains was supported. Among the exposure variables, exposure to suicidal communication was most prominent, suggesting that witnessing or receiving suicidal disclosures may play a disproportionately influential role in shaping clergy suicide prevention competency development. This finding aligns with the idea that encounters where a person openly expresses suicidal thoughts or intentions, rather than more distant, traumatic, or less interactive exposures, may be formative for skill acquisition and confidence-building (Mitchell et al., 2025). When exposed to suicidal communication, individuals can engage, interpret, and often respond in a way they consider most appropriate. In clergy suicide prevention training programs, simulated conversations, role plays, and structured opportunities to practice responding to suicidal communication may be more substantially beneficial than passive or observational learning. Conversely, the peripheral position of indirect and direct exposure to suicide attempt/death implies that more traumatic or emotionally intense exposures may not strongly translate into enhanced competencies, possibly because they may evoke distress or grief. The distinction points to the direction that not all exposures operate through the same mechanisms, calling for further research to investigate the possible mediators.

For current use of clergy suicide prevention competencies, the centrality of pastoral care with a suicidal person indicates that competencies grounded in relational engagement and supportive intervention may be structurally pivotal within the broader competency system. Apparently, the centrality of pastoral care may be connected to the limited exposure of the clergy in this study to formal training in suicide prevention encompassing the core competencies, yet interventions that strengthen pastoral care skills may produce cascading improvements across other competencies. If pastoral care serves as a dynamic driver of the connections in the competency network, strategically focusing on pastoral care with a suicidal person may be an integrative force to draw on knowledge, confidence, communication skills, and attitudinal openness for effective engagement in suicide prevention within faith communities.

### Strengths, Limitations, and Future Research Directions

Our study has several strengths, including the focus on an understudied, hard-to-reach sample of Nigerian Roman Catholic clergy; the use of validated, multidimensional suicide-related measures; and the examination of suicide prevention training as a moderating factor between exposure to suicide and domain-specific clergy use of suicide prevention competencies that could inform future professional development programs. Our study is also grounded in strong theoretical foundations, and we employed SEM and GGM to model complex relationships and uncover the underlying structure of interactions within the data. However, some limitations should be noted. First, the design of the study was cross-sectional which limits causal explanations of the associations among variables. Second, we assessed ‘ever’ received training using a single dichotomously scored item without considering recency of training and duration of training. Third, despite the substantial homogeneity across the eight dioceses in terms of culture, language, and church traditions, we did not conduct diocesan-level analyses due to the very small sample size in some of the dioceses. Future research should obtain longitudinal data, comprehensively assess training experiences, recruit larger samples to allow for diocesan-level analyses, and consider a mixed-methods design. Researchers should also examine mediator variables to clarify the pathways linking exposure to suicide with clergy use of suicide prevention competencies.

## Conclusion

To our knowledge, this is the first study to examine the association between the dimensions of lifetime exposure to suicide and the multifaceted current use of suicide prevention competencies. We further tested whether suicide prevention training moderates the relationships of different forms of lifetime exposure to suicide and the facets of current use of suicide prevention competencies. Our findings showed that while experiential exposure may contribute to the development of suicide prevention competencies among clergy, relying solely on experience may lead to uneven preparedness. Therefore, integrating structured suicide prevention training with pastoral experience may therefore be important for ensuring that priests possess consistent and comprehensive skills for responding effectively to suicidal individuals and supporting those affected by suicide. Another key finding of this study is the identification of aspects of suicide exposure (specifically, exposure to suicide communication) and the use of suicide prevention competencies (particularly, pastoral care with a suicidal person) that have the greatest structural influence in the model, indicating that experiential and skill-based domains may be especially valuable for enhancing clergy suicide prevention readiness and improving support for individuals at risk in faith communities.

## Supplementary Information

Below is the link to the electronic supplementary material.

Supplementary file1 (DOCX 67 kb)
