# Supplementary material for: Lifetime Exposure to Suicide and Current Use of Suicide Prevention Competencies Among Catholic Clergy in Nigeria: A Multidimensional Structural Analysis
Source: J Relig Health. 2026 Aug 20;65(5):4599–622. doi: 10.1007/s10943-026-02754-8 (PMC13649875; doi:10.1007/s10943-026-02754-8)
Supplement: Supplementary file 1 [file 10943_2026_2754_MOESM1_ESM.docx]

**Appendix**

**Table 6**

*Regularized Partial Correlation Matrix (Edge Weights) for the Network Analysis*

|  | **1** | **2** | **3** | **4** | **5** | **6** | **7** |
| --- | --- | --- | --- | --- | --- | --- | --- |
| 1. Suicidal Communication (ComEx) | — |  |  |  |  |  |  |
| 2. Indirect Exposure (IndEx) | .426 | — |  |  |  |  |  |
| 3. Direct Exposure (DirEx) | .478 | .000 | — |  |  |  |  |
| 4. Nonjudgemental Attitude (NonJd) | .000 | .000 | .000 | — |  |  |  |
| 5. Pastoral Care (Care) | .000 | .145 | .000 | .304 | — |  |  |
| 6. Confidence/Knowledge (Conf) | .000 | .000 | .000 | .000 | .431 | — |  |
| 7. Ministerial Approaches (Postv) | .000 | .000 | .000 | .000 | .282 | .276 | — |

Note. Values represent EBICglasso-regularized partial correlations; zeros indicate edges shrunk to zero. The Indirect Exposure–Pastoral Care edge (r = .145) is the primary cross-domain linkage.

**Figure 5**

*Case-dropping bootstrap stability plot for expected influence*

**Figure 6**

*Non-parametric bootstrap confidence intervals for edge weights*

**The Suicidal Behaviour Exposure Scale (SBX scale, Wolford-Clevenger et al., 2019, 2020)**

**Instructions**: The following items measure the frequency that clergy are exposed to those with suicidal behaviours. The numbers stand for: Never (1), Once in a while (2), Fairly often (3), Very often (4). **How often have you**:

| 1 | heard/seen someone joke about death by suicide? |  |  |  |  |
| --- | --- | --- | --- | --- | --- |
| 2 | heard/seen someone wish for death/suicide? |  |  |  |  |
| 3 | someone talked to you about their suicidal thoughts? |  |  |  |  |
| 4 | heard someone threaten to die by suicide? |  |  |  |  |
| 5 | heard about someone’s suicide attempt |  |  |  |  |
| 6 | heard specific details about suicide attempt |  |  |  |  |
| 7 | heard about someone dying from suicide? |  |  |  |  |
| 8 | been told specific details about someone’s dying by suicide |  |  |  |  |
| 9 | found someone after their suicide attempt |  |  |  |  |
| 10 | found someone’s body following suicide death |  |  |  |  |
| 11 | witnessed someone’s suicide attempt |  |  |  |  |
| 12 | witnessed someone’s death by suicide |  |  |  |  |
| 13 | found someone’s poetry/diary about death/suicide |  |  |  |  |
| 14 | found someone’s suicide note |  |  |  |  |
| 15 | witnessed someone plan/prepare for suicide |  |  |  |  |
| 16 | tried to stop someone’s attempt |  |  |  |  |

**Measure of Suicide Prevention Clergy Competencies (MSPCC, Mason et al., 2020).**

**Instructions**: Please indicate the extent to which you practice this skill in your current faith community. If you have never talked to a suicidal person or a friend or family member after a suicide, choose N/A. Response scale: never/not like me (0), rarely (1), sometimes/somewhat like me (2), often (3), always/like me (4), not applicable (NA).

| 1 | When a suicidal person talks to me, I am nonjudgmental |  |  |  |  |  |
| --- | --- | --- | --- | --- | --- | --- |
| 2 | When a person who has attempted suicide talks to me, I am nonjudgmental |  |  |  |  |  |
| 3 | When a survivor talks to me after his/her friend or family member’s suicide  death, I am nonjudgmental |  |  |  |  |  |
| 4 | When a suicidal person talks to me, I take her or his culture into account (e.g., is the suicidal person experiencing discrimination?) |  |  |  |  |  |
| 5 | I have reflected on my theology of suicide. |  |  |  |  |  |
| 6 | When a suicidal person talks to me, I am clear about my role as a spiritual leader not a mental health professional. |  |  |  |  |  |
| 7 | When a suicidal person talks to me, my first goal is to listen. |  |  |  |  |  |
| 8 | When a suicidal person talks to me, I am confident about next steps. |  |  |  |  |  |
| 9 | When a suicidal person talks to me, I know the right questions to ask. |  |  |  |  |  |
| 10 | When a suicidal person talks to me, I know where to refer them for help |  |  |  |  |  |
| 11 | When a suicidal person talks to me, I know how to reach out for support for myself if needed. |  |  |  |  |  |
| 12 | In my faith community, the congregation watch over each other |  |  |  |  |  |
| 13 | When a suicide death happens, I know how to care for the friend(s) and family member(s)/the survivor(s). |  |  |  |  |  |
| 14 | When a suicide death happens, I know how to conduct a funeral that is helpful to survivor(s) and congregant(s). |  |  |  |  |  |
| 15 | During a suicide funeral, I know how to strike a balance between talking about suicide without making it sound like a good solution to life’s problems. |  |  |  |  |  |
| 16 | When a suicide death happens, I do not take on guilt. |  |  |  |  |  |
| 17 | I can talk to my congregation (preach or teach) about suicide. |  |  |  |  |  |
